# Advances in targeted therapy and biomarker research in thyroid cancer

**DOI:** 10.3389/fendo.2024.1372553

**Published:** 2024-03-04

**Authors:** Mei Guo, Yuqi Sun, Yuyao Wei, Jianxin Xu, Chun Zhang

**Affiliations:** ^1^ School of Nursing, Nanjing University of Chinese Medicine, Nanjing, China; ^2^ School of Pharmacy, Nanjing University of Chinese Medicine, Nanjing, China; ^3^ Affiliated Hospital of Nanjing University of Chinese Medicine, Nanjing, China

**Keywords:** thyroid cancer, targeted therapy, biomarker research, disease treatment, clinical application

## Abstract

Driven by the intricacy of the illness and the need for individualized treatments, targeted therapy and biomarker research in thyroid cancer represent an important frontier in oncology. The variety of genetic changes associated with thyroid cancer demand more investigation to elucidate molecular details. This research is clinically significant since it can be used to develop customized treatment plans. A more focused approach is provided by targeted therapies, which target certain molecular targets such as mutant BRAF or RET proteins. This strategy minimizes collateral harm to healthy tissues and may also reduce adverse effects. Simultaneously, patient categorization based on molecular profiles is made possible by biomarker exploration, which allows for customized therapy regimens and maximizes therapeutic results. The benefits of targeted therapy and biomarker research go beyond their immediate clinical impact to encompass the whole cancer landscape. Comprehending the genetic underpinnings of thyroid cancer facilitates the creation of novel treatments that specifically target aberrant molecules. This advances the treatment of thyroid cancer and advances precision medicine, paving the way for the treatment of other cancers. Taken simply, more study on thyroid cancer is promising for better patient care. The concepts discovered during this investigation have the potential to completely transform the way that care is provided, bringing in a new era of personalized, precision medicine. This paradigm shift could improve the prognosis and quality of life for individuals with thyroid cancer and act as an inspiration for advances in other cancer types.

## 1 Introduction

Thyroid cancer originates from the thyroid gland, a butterfly-shaped organ located near the base of the neck (1). There has been a rise in its occurrence during the last several years (2). Thyroid cancer risk factors include radiation exposure, particular genetic abnormalities, and a family history of the disease (3). Women are more likely than men to develop thyroid cancer, and the risk increases with age (3). Some regions with high iodine intake might have a higher prevalence of subtypes of thyroid cancer (4). Possible symptoms include a lump or swelling in the neck, swallowing difficulty, chronic neck pain, and hoarseness (4). The diagnosis procedure involves a physical examination, imaging tests including ultrasonography, and a biopsy to confirm the existence of cancerous cells (4). The potential treatment options are determined by the type and stage of thyroid cancer. Surgically removing the thyroid gland is a common approach, and any remaining cancer cells are treated with radioactive iodine therapy (5). Thyroid cancer usually has an excellent prognosis, especially if it is detected early and treated promptly (6). Regular follow-up treatment is crucial to monitor for any signs of a recurrence (6).

Molecular biomarkers are essential for thyroid cancer diagnosis, prognosis, and treatment because they offer important information on the molecular features of the illness (7). BRAF mutation (8), RAS mutation (9), RET/PTC rearrangement (10), PAX8/PPARγ rearrangement (11), and TERT promoter mutation (12), are among the major molecular indicators linked to thyroid cancer. The mutation V600E in the BRAF gene is frequently found in papillary thyroid carcinoma (PTC). Identifying this mutation improves prognosis and may have an impact on therapy choices (8). Thyroid tumors that are poorly differentiated and follicular frequently include mutations in the RAS gene. Detecting RAS mutations aids in improving the precision of diagnosis and forecasting the behavior of tumors (9). PTC is commonly associated with RET proto-oncogene rearrangements, such as RET/PTC. These reorganizations function as crucial indicators for diagnosis (10). A particular genetic change that is present in follicular thyroid carcinoma (FTC) and helps distinguish this thyroid cancer subtype from other types of thyroid cancer is the PAX8-PPARγ rearrangement (11). A worse prognosis is linked to aggressive forms of thyroid cancer that have mutations in the TERT promoter region. Risk categorization is informed by the discovery of TERT mutations (12). Comprehending these molecular biomarkers facilitates more accurate diagnosis, prognostication, and customized treatment plans for individuals with thyroid cancer.

Thyroid cancer targeted treatment aims to more precisely interfere with the development and multiplication of cancer cells using specialized ways to target molecules involved in cancer growth (13). Inhibitors of tyrosine kinase (14), inhibitors of thyroid hormone receptor (15), radioactive iodine treatment (16), immunotherapy (17), and gene targeted therapy (18) are some of the main targeted treatments for thyroid cancer. Drugs such as Sorafenib and Lenvatinib target abnormal activation of tyrosine kinase in thyroid cancer. These drugs prevent the enzyme from functioning, which hinders the development and division of cancer cells (14). Drugs such as Dabrafenib and Trametinib disrupt certain signaling pathways in malignant cells to treat unresectable or recurrent thyroid cancer (15). Despite difficulties during surgery or recurrence, radioactive iodine therapy continues to be efficient in eliminating any residual cancer cells using radioactive iodine (16). To treat resistant thyroid cancer, immune checkpoint inhibitors such as pembrolizumab and nivolumab are being studied. These inhibitors work by stimulating the immune system against cancer cells (17). To precisely restrict the proliferation of cancer cells, novel medications that specifically target gene abnormalities have been produced (18). Customized to the specific needs of each patient and kind of pathology, targeted treatment requires medical oversight to prevent adverse effects and maintain maximum effectiveness and quality of life.

Biomarker research and targeted therapeutic research offer unprecedented opportunities for the clinical treatment of thyroid cancer, but also present formidable challenges. In this article, we comprehensively analyze the conventional treatment strategies for thyroid cancer, the status of biomarker research, and the latest developments in targeted therapy.

## 2 Overview of thyroid cancer

### 2.1 Pathological features of thyroid cancer

Due to its heterogeneous nature, thyroid carcinoma exhibits a wide range of clinical features (19). Thyroid cancer’s early asymptomatic state is one of its distinctive features. Many people don’t know they have the disease unless they happen to feel thyroid nodules while getting regular checkups or imaging tests (19). Even though the majority of thyroid nodules are benign, an in-depth examination is necessary to rule out cancer (19). These nodules can be solitary or multi-located, and they can differ in size and consistency (20). A crucial diagnostic technique for determining these nodules and differentiating between benign and malignant lesions is fine-needle aspiration (FNA) biopsies (20). Thyroid imaging is a crucial approach for thyroid cancer diagnostics. Comprehensive details regarding nodule features, such as size, composition, and vascularity, can be obtained using ultrasonography (20). Computed tomography (CT) and magnetic resonance imaging (MRI) provide information on the degree of tumor invasion and the involvement of nearby structures, which helps with surgical planning (21). In cases of differentiated thyroid cancer, radioiodine scintigraphy is used to measure the thyroid tissue’s uptake of radioactive iodine, which helps guide postoperative care and recurrence surveillance (21). In situations where iodine uptake is restricted, PET scans can be employed to obtain further insights into the dissemination of the disease.

Thyroid hormone levels can fluctuate in certain individuals, which may lead to symptoms like exhaustion, mood swings, and changes in weight (22). But it is important to remember that thyroid cancer can coexist with normal thyroid function, so a diagnosis based solely on hormonal alterations is inadequate (22). Clinical signs such as neck pain, discomfort, or a lump sensation may indicate an individual needs medical attention. Thyroid nodule expansion or involvement of other structures frequently causes these symptoms (23). Swallowing difficulties and hoarseness can arise from tumor compression or infiltration of the esophagus or recurrent laryngeal nerve. Thyroid cancer frequently presents with the enlargement of the neck lymph nodes (23). Palpable lymph nodes, especially those exhibiting questionable features, require further imaging examinations and biopsies to assess the degree of disease dissemination (24). There is histological variation in thyroid cancer, with PTC being the most common subtype. Under a microscope, PTC frequently exhibits well-differentiated appearance with distinctive nuclear characteristics (25). Another histological subtype that has unique features and is linked to an increased risk of vascular invasion is called FTC (25). Despite being uncommon, one aggressive type of thyroid cancer known for its quick growth and dismal prognosis is anaplastic thyroid carcinoma (ATC). Parafollicular C cells are the source of medullary thyroid cancer (MTC), which has been linked to family disorders.

### 2.2 Health hazards of thyroid cancer

It is imperative that patients, healthcare providers, and the public comprehend the various consequences associated with thyroid cancer. First and foremost, thyroid cancer has the potential to disrupt the delicate hormone balance, which might affect the body’s metabolism and energy regulation (26). These hormones’ dysregulation, which is frequently observed in thyroid cancer cases, can cause symptoms like weariness, worry, emotional instability, and variations in weight (26). Thyroid cancer can present with physical symptoms up to the neck, where the primary tumor is located (27). It is possible for patients to feel pressure, discomfort, or pain in their necks. As the tumor grows larger, it may compress nearby structures and cause symptoms like hoarseness and difficulty swallowing (27). These physical symptoms lead to a lower quality of life in addition to interfering with daily activities (27). Thyroid cancer frequently involves lymph nodes, particularly if there is cervical lymphadenopathy (28). Palpable swelling, pain, and an increased level of intricacy in the disease can all result from enlarged lymph nodes in the neck. Thyroid cancer cells have the potential to spread throughout the circulation and cause distant metastases in organs including the lungs, bones, or other vital organs (28). The health risks are increased when tumor metastasis occurs, making treatment more difficult and raising the possibility of unfavorable results. The health risks are especially significant for some subtypes of thyroid cancer, such as ATC.

Aggressive growth, quick development, and a bleak prognosis are characteristics of ATC. The seriousness of the health hazards associated with specific thyroid cancer subtypes is highlighted by the fact that patients with ATC frequently encounter major hurdles regarding treatment response and overall survival (29). There are potential health risks associated with the diagnostic and therapy procedures itself. There is an inherent risk of problems with diagnostic tests, such as hemorrhage, infection, and injury to adjacent structures (29). These hazards include imaging examinations, surgical treatments, and fine-needle aspiration (FNA) biopsies (30). Furthermore, radiation induced malfunction of the salivary glands and its long-term repercussions are possible side effects and problems of radioactive iodine ablation, a popular postoperative treatment for thyroid cancer (31). Moreover, it is impossible to overstate the psychological effects of thyroid cancer (31). Receiving a cancer diagnosis is a transformative experience that frequently results in psychological anguish, anxiety, and despair. Individuals may struggle with uncertainty anxiety, worries about how their treatments will turn out, and the psychological weight of knowing they may die (32). A complete approach to patient care for thyroid cancer must address the psychological elements of the disease (32). Thyroid cancer has consequences for society and the economy in addition to personal costs. The financial burden on individuals and healthcare systems is exacerbated by the expenses related to thyroid cancer diagnosis, treatment, and long-term management (33). Furthermore, there are wider societal ramifications from the possible loss of productivity and quality of life for those impacted (33). The health risks associated with thyroid cancer are multifaceted, involving physical, physiological, and psychological factors.

### 2.3 Conventional treatment strategies for thyroid cancer

Surgical techniques, radioactive iodine therapy, and thyroid hormone replacement are commonly employed as conventional therapeutic procedures for thyroid cancer (34). Comprehending these therapeutic approaches is essential to maximizing results and guaranteeing the welfare of patients with thyroid cancer (34). The mainstay of treatment for thyroid cancer is surgery, which aims to remove the tumor and any nearby lymph nodes that may need to be removed (35). Tumor size, metastasis presence, and histological subtype all influence whether surgery is performed (35). Thyroidectomy is the most frequent surgical surgery in which the thyroid gland is removed whole or in part (36). For differentiated thyroid tumors, radioactive iodine treatment is frequently used after surgery (36). Radioactive iodine is used in this therapy to specifically target and eliminate tiny cancer cells and any surviving thyroid tissue (36). This therapy approach is quite particular because thyroid cells, especially malignant ones, have a special capacity to absorb iodine (36). A crucial part of postoperative treatment is thyroid hormone replacement medication (37). Individuals need to take thyroid hormone supplements for the rest of their lives to maintain normal physiological processes since thyroidectomy causes the loss of thyroid function (37). The most often recommended drug is levothyroxine, which is a synthetic version of the thyroid hormone thyroxine T4 (37). External beam radiation therapy may be an option for individuals whose aggressive or advanced thyroid tumors do not respond to standard care (37). The goal of this treatment is to stop the growth of the malignant tissues by applying specific radiation. Usually, external beam radiation is saved for situations in which radioactive iodine therapy and surgery are not enough to manage the illness. Advances in thyroid cancer therapy options are mostly dependent on clinical studies. These clinical studies assess the safety and effectiveness of new medications, combinations of treatments, and therapy modalities. Enrolling in clinical trials provides patients with access to novel treatments that may result in better outcomes (38). A comprehensive approach to therapy is ensured by the collaboration of endocrinologists, surgeons, radiation oncologists, and medical oncologists in customizing treatment programs depending on the diagnosis of each patient (38). Tracking therapy response and identifying any recurrence signals requires routine monitoring with imaging scans, blood tests, and clinical evaluations (38). In summary, radioactive iodine therapy, surgery, and thyroid hormone replacement are traditional treatments for thyroid cancer. These interventions, which are based on the unique features of the tumor, are designed to get rid of cancerous cells, control symptoms, and stop the tumor from coming back.

## 3 Advances in biomarker research of thyroid cancer

**Figure 1:**
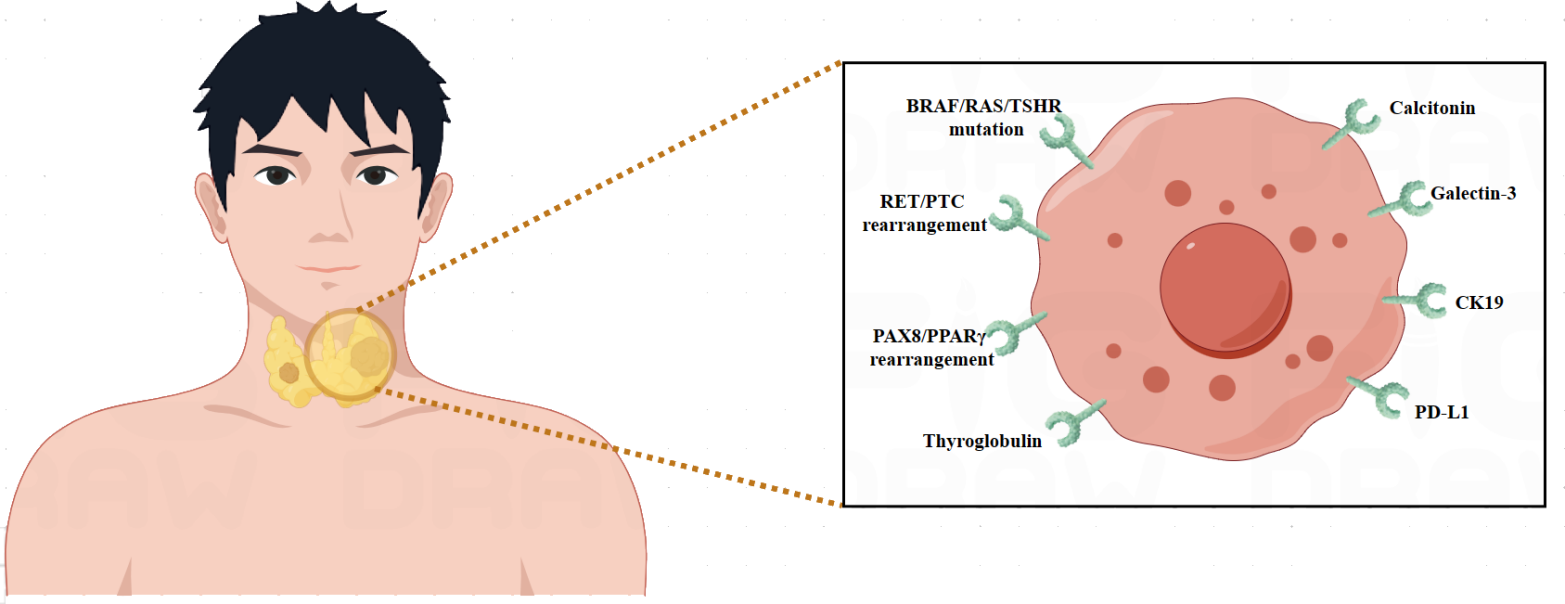
Overview of biomarkers for thyroid cancer.

Thyroid cancer molecular biomarker research has made great progress, providing important insights into the disease’s complexity (7). Proteomics and genomics advances have revealed particular genetic abnormalities and molecular changes linked to distinct subtypes of thyroid cancer (7). Markers such as BRAF and RAS mutations have become critical for diagnosis and prognosis (8, 9). Furthermore, studies have shown intriguing biomarkers that indicate therapy response, which might help with treatment selection. By evaluating circulating tumor DNA, liquid biopsies provide a non-invasive way to track the course of the illness and identify recurrences before they become severe (39). Clinical practice may greatly benefit from the integration of molecular profiling in order to improve patient outcomes, risk classification, and customized treatment plans. The field of thyroid cancer research is being shaped by ongoing studies into new biomarkers, which are helping to better understand the condition and opening the way for innovative targeted therapies (**Table 1**) (**Figure 1**).

**Table 1:** Biomarker research of thyroid cancer.

Biomarkers	Notes	Diseases	Drugs	Functions	References
BRAF	V600E mutation	PTC	Vemurafenib, Dabrafenib	Activation of MAPK pathway	(40–42)
RAS	RAS mutation	FTC	Sotorasib	Cell growth and differentiation	(43–47)
TSHR	TSHR mutation	PTC, FTC	Protirelin	Activation of the receptor	(22, 48–52)
RET	RET/PTC rearrangement	PTC	Vandetanib Cabozantinib	Cell survival and differentiation	(10, 53–55)
PAX8/PPARγ	PAX8/PPARγ rearrangement	FTC	Pioglitazone	Lipid metabolism	(11, 56–58)
TERT	TERT promoter mutations	ATC	Under investigation	Cell division	(59–61)
miR-146b	Downregulation	PTC	Under investigation	Tumor characteristics	(63)
miR-221/222	Upregulation	PTC, ATC	Under investigation	Cell proliferation and survival	(64)
miR-21	Upregulation	PTC	Under investigation	Cell proliferation and migration	(62)
miR-29b	Downregulation	PTC, FTC	Under investigation	Modulation of ECM components	(62)
miR-96	Upregulation	PTC, FTC	Under investigation	Cell proliferation and migration	(62)
Thyroglobulin	Upregulation	PTC, FTC	Under investigation	thyroid hormone biosynthesis	(66–69)
Calcitonin	Upregulation	MTC	Under investigation	Calcium homeostasis	(70–74)
Galectin-3	Upregulation	PTC	Under investigation	Cell adhesion and apoptosis	(75–80)
CK19	Upregulation	PTC	Under investigation	Tumor development and progression	(81–87)
ctDNA	Upregulation	PTC, FTC	Under investigation	Detection of specific genetic alterations	(88–91)
PD-L1	Upregulation	PTC, FTC	Atezolizumab Durvalumab	Immunotherapy	(92–96)

### 3.1 Genetic mutations

The BRAF mutation is a critical genetic modification in the landscape of thyroid cancer, notably in PTC (8). The substitution of glutamic acid for valine at codon 600 (V600E) is the most common BRAF mutation linked to thyroid cancer (8). The BRAF V600E mutant is present in a considerable number of PTC patients, which emphasizes its utility as a molecular biomarker (8). This mutation causes the MAPK pathway to become constitutively activated, which promotes unchecked cell division and growth. The disease development and severity of PTC are influenced by the downstream effects of this abnormal signaling cascade (40). The existence of the BRAF V600E mutant in clinical settings has consequences for both diagnosis and prognosis (40). It helps distinguish between benign thyroid nodules and malignant tumors, making it a useful diagnostic marker. Moreover, its discovery in PTC is linked to particular clinicopathological characteristics such lymph node metastases, extrathyroidal extension, and heightened recurrence risk (41). The BRAF V600E mutation has become a target for innovative therapeutic approaches. Preclinical and clinical research have demonstrated the promise of inhibitors like vemurafenib and dabrafenib, which are specially made to target mutant BRAF proteins (41). These BRAF inhibitors stop the aberrant signaling cascade, which inhibits the growth of tumors and may open up new therapeutic options that are more focused and efficient (42). Comprehending the molecular details of the BRAF mutation has improved our understanding of the biology of thyroid cancer and opened the door to tailored treatment approaches. Treatment for thyroid cancer has changed dramatically with the introduction of targeted medications intended to block the effects of the BRAF V600E mutation.

RAS mutations are a key molecular change associated with thyroid cancer and specific forms of poorly differentiated and anaplastic thyroid carcinomas (9). RAS mutations are often linked to FTC in thyroid cancer, where they have a role in the development and course of the illness (43). Although these mutations are less common in well-differentiated PTC, they can still be detected in certain instances, particularly in those that have a follicular variation (44). The presence of RAS mutations frequently indicates a different molecular route in thyroid carcinogenesis than the more common BRAF-mutated PTC (44). RAS mutations have distinct clinical effects on thyroid carcinoma. RAS-mutant FTC frequently has a follicular growth pattern and may be linked to vascular invasion, which may increase the tumor’s aggressiveness (45). By contrast, PTC with RAS mutations may exhibit unique histological characteristics, such as a follicular growth pattern, necessitating a correct diagnosis in order to implement the best course of treatment (46). The precise prognostic significance is still unclear despite several studies suggesting a relationship with more aggressive disease features, such as an increased risk of recurrence and a reduced overall survival rate (46). RAS mutations present therapeutic problems since they have demonstrated to be less responsive to targeted therapy than other variants, such as BRAF V600E. The deficiency of efficacious targeted inhibitors exclusively for cancers mutant in RAS highlights the necessity for substitute therapeutic approaches (47). In addition to improving diagnostic precision, knowledge of the molecular landscape of RAS mutations in thyroid cancer may influence therapy choices. Given the unique biological activity linked to RAS mutations, the discovery of these mutations may lead to increased surveillance and customized treatment strategies.

Thyroid stimulating hormone receptor (TSHR) mutations are genetic changes that affect the function of the TSHR gene, which is an important regulator of thyroid function (48). Thyroid function dysregulation can arise from TSHR mutations that either constitutively activate or inactivate the receptor (48). Depending on the type of mutation, these mutations can cause both hypothyroidism and hyperthyroidism, among other thyroid problems (49). Increased sensitivity to TSH as a result of constitutive activation of the TSHR mutation results in unregulated thyroid hormone (49). This situation is frequently linked to hyperthyroidism, including Graves’ disease, an autoimmune condition in which autoantibodies activate TSHR (49). Conversely, mutations that render the TSHR inactive may result in a decreased thyroid hormone synthesis via decreasing response to TSH (50). This disorder is linked to hypothyroidism and may be a factor in thyroid dyshormonogenesis or congenital hypothyroidism (50). The particular mutation and its functional ramifications determine the clinical significance of TSHR mutations (51). When hypothyroidism is present, symptoms like lethargy, weight gain, and cold sensitivity may appear, whereas symptoms of hyperthyroidism may include anxiety, accelerated heart rate, and weight loss (51). Genetic testing can detect TSHR mutations, which is useful information for a precise diagnosis and for informing therapy choices (22). Management approaches for patients of hyperthyroidism may involve radioactive iodine treatment, antithyroid drugs, or surgery (22). Thyroid hormone replacement medication is commonly used to treat hypothyroidism brought on by TSHR mutations. Not only is it essential for clinical care to comprehend the genetic basis of TSHR mutations, but it also advances thyroid biology research (52). Our understanding of thyroid problems is improved by elucidating the molecular pathways behind these alterations, which opens the door to customized treatment methods and targeted medicines (52). The precise form of the mutation determines the clinical presentation and therapeutic approaches, highlighting the need of genetic testing in the all-encompassing care of thyroid problems.

### 3.2 Gene rearrangements

The RET/PTC rearrangement is a genetic change that contributes to the development of papillary thyroid carcinoma, the most frequent kind of thyroid cancer (10). This rearrangement includes the fusing of the RET proto-oncogene with the PTC gene, which results in a chimeric gene that promotes uncontrolled cell growth and proliferation (10). The fusion event in the setting of RET/PTC rearrangements results in a constitutively active RET kinase, which in turn stimulates downstream signaling pathways, most notably the MAPK pathway (10). The most prevalent fusion forms, with unique clinicopathological characteristics, are RET/PTC1 and RET/PTC3 (10). These reorganizations are linked to a particular histological pattern that has a noticeable papillary form (10). Finding RET/PTC rearrangements is important from a diagnostic standpoint. The existence of these rearrangements contributes to a more precise tumor categorization by helping to differentiate PTC from other thyroid lesions (53). Furthermore, risk assessment and treatment planning may be impacted by the discovery of RET/PTC rearrangements (53). Certain clinicopathological characteristics, such as a greater frequency in younger individuals and a higher probability of multifocality, are frequently seen in RET/PTC-positive tumors (54). The correlation with radiation exposure, especially during early life, emphasizes the environmental elements influencing the formation of these rearrangements (54). One therapeutic approach that may be used is to address the abnormal RET kinase activity. Tyrosine kinase inhibitors that target RET, such as vandetanib and cabozantinib, have showed promise in clinical studies, while particular inhibitors for RET/PTC-positive thyroid tumors are currently being investigated (54). By blocking the signaling pathways started by RET/PTC rearrangements, these targeted treatments hope to slow the growth of tumors and maybe improve the prognosis of patients who carry these particular genetic abnormalities (55). A specific molecular characteristic of PTC that adds to our knowledge of the disease’s pathophysiology is the presence of RET/PTC rearrangements (55). Their detection has consequences for both diagnosis and prognosis, and continued research into targeted therapies might lead to more efficient treatments specifically designed for patients with thyroid malignancies that are positive for RET or PTC (55). As molecular insights emerge, deciphering the intricacies of these rearrangements holds promise for enhancing precision medicine in thyroid cancer treatment.

The PAX8/PPARγ rearrangement is a molecular abnormality found in thyroid carcinoma, especially FTC (11). The fusion of PAX8 and PPARγ genes creates a chimeric gene with carcinogenic potential (11). The follicular variety of FTC is characterized by the PAX8/PPARγ rearrangement, which offers molecular insights into the pathophysiology of this subtype (11). Although FTC is thought to be less aggressive than PTC, its ability to spread to other areas of the body and invade blood vessels presents management issues that require a fuller knowledge of the molecular basis of the disease (56). Accurate diagnosis of FTC is aided clinically by identifying the PAX8/PPARγ rearrangement (56). For the purpose of choosing the best course of treatment, it is essential to distinguish between FTC and follicular adenoma, a benign thyroid lesion that shares a similar histological appearance (57). Molecular testing is frequently used to validate the existence of this rearrangement, which helps to provide a more accurate classification of thyroid cancers (57). Histologically, FTCs that are positive for PAX8/PPARγ may display distinct characteristics including a solid or trabecular development. Furthermore, treatment planning and risk stratification may be affected by the discovery of this rearrangement (58). Treatment with radioactive iodine may be taken into consideration when there is vascular invasion or a higher chance of recurrence. Future targeted therapeutics targeting the PAX8/PPARγ fusion protein could result in more precise and efficacious interventions (58). A unique molecular characteristic linked to follicular thyroid cancer is the rearrangement of PAX8/PPARγ (58). Its detection guides the management of individuals with this particular genetic mutation and has diagnostic and potentially prognostic ramifications. There is hope for the development of targeted treatments that could improve treatment options and outcomes for those with PAX8/PPARγ-positive thyroid malignancies as research into these complex tumors continues.

### 3.3 TERT promoter mutations

Mutations in the telomerase reverse transcriptase (TERT) gene promoter area are linked to worse prognoses and more aggressive forms of thyroid cancer (12). TERT promoter mutations are genetic changes that take place in the telomerase reverse transcriptase promoter, which is the promoter region of the TERT gene (12). These mutations are linked to increased telomerase activity, which promotes cellular immortalization and aids in oncogenesis (12). TERT promoter mutations occur frequently in aggressive subtypes of thyroid cancer, including versions of differentiated thyroid carcinoma (12). These alterations are linked to more advanced stages of the disease, larger tumors, and an increased risk of lymph node metastases and extrathyroidal extension (59). Furthermore, TERT promoter mutations are linked to a worse chance of survival and recurrence of the disease, making them a predictor of poor prognosis (59). Finding TERT promoter mutations has important diagnostic and prognostic implications in the clinical setting. Their existence may suggest a more severe disease phenotype in thyroid cancer diagnosis, impacting postoperative care and treatment choices (60). When a tumor is difficult to distinguish based just on histopathological findings, TERT promoter mutation analysis is very pertinent (60). Therapeutic treatment planning may be affected by the presence of TERT promoter mutations. The discovery of TERT promoter mutations may lead to more careful monitoring and individualized treatment strategies, as tumors containing these mutations may show resistance to traditional medicines (61). Nevertheless, there are currently few readily accessible targeted treatments that directly suppress mutations in the TERT promoter. Researching the molecular effects of TERT promoter mutations may have therapeutic applications in addition to improving thyroid cancer diagnosis and prognostication (61). TERT promoter mutation analysis may eventually be included into standard clinical practice, which could improve the accuracy of managing thyroid cancer and open up new possibilities for individualized treatment plans.

### 3.4 microRNA expression profiles

Dysregulation of microRNA (miRNA) expression is observed in thyroid cancer. Particular miRNA patterns are linked to the development, spread, and response to therapy of tumors (62). The complex molecular signatures known as miRNA expression profiles offer important new perspectives on the cellular mechanisms governing gene expression (62). MiRNA expression profiles have become important resources in the field of cancer research, particularly thyroid cancer, since they provide insight into the etiology, prognosis, and possible therapeutic approaches of the illness (62). A typical characteristic of cancer is the dysregulation of miRNAs, whose abnormal expression aids in the development, advancement, and metastasis of malignancies (62). Different histological subtypes of thyroid cancer have been found to have diverse miRNA expression profiles, which helps with the molecular classification of malignancies (62). miR-146b is known for its ability to modulate inflammation, and it is frequently downregulated in PTC (63). The miR-221/222 cluster is often overexpressed in thyroid cancer and has a role in controlling important signaling pathways that support the growth and survival of cells (64). Thyroid cancer is associated with an upregulation of miR-155, which facilitates the migration and proliferation of cancer cells (65). Higher expression of miR-21 has been linked to a number of malignancies, including thyroid carcinoma (62). It encourages cell invasion and survival, which adds to the aggressive nature of thyroid cancers (62). Tumor suppressor miR-34a is frequently downregulated in thyroid carcinoma. Advanced stages of the disease are linked to its decreased expression (62). MiR-29b downregulation has been linked to aggressive thyroid carcinoma characteristics. Its functions include suppressing metastasis and modifying the components of the extracellular matrix (62). MiRNA expression profiles function as predictive and diagnostic indicators. Preoperative diagnosis accuracy can be improved by using specific miRNA signatures that can distinguish between benign and malignant thyroid lesions (62–64). MiRNA expression patterns have a wide range of potential therapeutic applications. To restore or suppress certain miRNAs for therapeutic purposes, researchers are investigating the creation of miRNA-based therapies, such as miRNA mimics and inhibitors (62–64). Technological developments like microarray studies and high-throughput sequencing have made it possible to profile miRNAs in a wide range of biological samples (62–64). These methods enable researchers to find important regulatory networks, discover patterns of global miRNA expression, and locate putative illness biomarkers (62–64). MiRNA expression profiles provide a complex layer of biological data that aids our knowledge of thyroid cancer and other disorders (62–65). The complex interaction between miRNAs and gene regulation sheds light on the molecular landscape of malignancies, opening up new diagnostic, prognostic, and therapeutic prospects. As research advances, incorporating miRNA expression profiling into clinical practice has the potential to improve the accuracy of thyroid cancer care and advance customized therapy methods.

### 3.5 Thyroglobulin

Thyroglobulin is a protein generated by both healthy and malignant thyroid cells (66). Serum thyroglobulin levels are measured as a biomarker of recurrence following thyroidectomy (66). Elevated levels might suggest persistent or recurring illness. Thyroglobulin is generated and maintained in thyroid follicular cells, where it is essential in the intricate process of thyroid hormone production (66). Thyroglobulin is a big protein with a molecular weight of about 660 kDa. It consists of a single polypeptide chain with numerous tyrosine residues that are iodinated during thyroid hormone production (66). Thyroglobulin is synthesized in the endoplasmic reticulum of thyroid follicular cells before being transferred to the thyroid follicular colloid, where thyroid hormone is synthesized (67). Thyroglobulin’s principal role is to act as a framework for thyroid hormone production and storage, namely thyroxine (T4) and triiodothyronine (T3). Thyroid peroxidase enzymes iodize particular tyrosine residues on thyroglobulin, resulting in monoiodotyrosine (MIT) and diiodotyrosine. These iodinated tyrosine residues are coupled together to create T3 and T4 inside the thyroglobulin structure (67). When triggered by thyroid-stimulating hormone (TSH), thyroid follicular cells transport thyroglobulin from the colloid to vesicles. Enzymes in these vesicles breakdown thyroglobulin, releasing T3 and T4 into the circulation. Thyroglobulin is also secreted into the bloodstream, acting as a quantifiable indication of thyroid function. Serum thyroglobulin levels are an important clinical tool for assessing thyroid diseases (68). Thyroglobulin levels are typically modest in healthy people, but increased levels might suggest a variety of diseases. Following thyroidectomy for thyroid cancer, serum thyroglobulin levels are measured as a tumor marker (68). An increase in thyroglobulin levels might indicate remaining or recurring thyroid cancer cells. Thyroglobulin testing is also used to determine the efficacy of thyroid cancer therapies such radioactive iodine therapy (68). While thyroglobulin is an important clinical marker, measuring it might be hindered by anti-thyroglobulin antibodies (TgAbs). This can interfere with reliable thyroglobulin tests, necessitating cautious interpretation in clinical situations (68). Thyroglobulin is a key glycoprotein that plays an important role in thyroid hormone production and storage. Its blood levels give useful information for measuring thyroid function, treating thyroid diseases, and tracking thyroid cancer therapy results. The intricate interaction of thyroglobulin inside the thyroid gland emphasizes its importance in maintaining hormonal balance and general thyroid function.

### 3.6 Calcitonin

Calcitonin is an important biomarker in the setting of thyroid cancer, particularly in the diagnosis and monitoring of MTC (69). MTC is an uncommon form of thyroid cancer that arises from parafollicular C cells, accounting for just 1-2% of all thyroid malignancies (70). Calcitonin is a hormone generated by the thyroid gland’s C cells. Its principal physiological duty is to maintain calcium homeostasis by reducing osteoclast activity in bones, resulting in less calcium release into the circulation (70). Calcitonin levels are considerably higher in MTC patients (71). Unlike other types of thyroid cancer, where thyroglobulin is the predominant tumor marker, calcitonin is highly unique to MTC, making it an important diagnostic tool for this subtype (71). Serum calcitonin levels can help in early identification and diagnosis of MTC. Elevated calcitonin levels, particularly in the baseline state and after stimulation tests, indicate the existence of MTC (72). Calcitonin is used as a biomarker not just for initial diagnosis, but also to detect residual or recurrent illness following surgery (72). Calcitonin levels at baseline in MTC patients can be used to predict prognosis (72). Higher preoperative calcitonin levels are frequently linked with more advanced disease stages, which can impact treatment options and postoperative care (73). Calcitonin can be released via stimulation tests such as the calcium or pentagastrin stimulation test. The extent of the calcitonin rise in response to these tests reveals more about the MTC’s functioning and aggressiveness (73). Following surgical thyroid gland removal in MTC patients, blood calcitonin levels must be monitored to determine therapy success and identify illness recurrence (73). Persistent or rising calcitonin levels during follow-up may suggest residual or recurrent MTC, necessitating further imaging examinations and treatment measures (74). Given its prominent involvement in MTC, calcitonin has been investigated as a possible treatment target. Some medications try to reduce calcitonin synthesis and secretion, providing a more tailored approach to treating MTC (74). Calcitonin is a key biomarker for medullary thyroid cancer. Its assessment is critical in the diagnosis, monitoring, and prognosis of MTC, emphasizing its clinical importance in the overall therapy of this rare but specific kind of thyroid cancer (74). Current investigation seeks to improve understanding of calcitonin’s role and its potential as a therapeutic target in medullary thyroid cancer.

### 3.7 Galectin-3

Belonging to the galectin family of β-galactoside-binding proteins, galectin-3 is important when it comes to thyroid cancer, especially when it comes to the diagnosis and prognosis of thyroid nodules (75). This multifunctional protein participates in a variety of biological activities, including cell adhesion, proliferation, differentiation, and death (75). Galectin-3 has emerged as an important biomarker in thyroid cancer, with implications for risk classification and treatment decisions (75). Galectin-3 is significantly overexpressed in thyroid cancer, especially in PTC, one of the most frequent kinds of thyroid cancer (76). The level of expression is insufficient or absent in normal thyroid tissue (76). Galectin-3 immunohistochemistry has proven to be an effective method for discriminating between benign and malignant thyroid nodules (76). High galectin-3 expression indicates malignancy, which aids in the preoperative evaluation of thyroid nodules (77). Galectin-3 expression is linked to more aggressive characteristics in thyroid cancer (77). High galectin-3 levels have been associated to increased tumor growth, extrathyroidal extension, and lymph node metastasis in PTC (78). Incorporating galectin-3 testing into diagnostic algorithms helps stratify the risk of malignancy in thyroid nodules, aiding doctors in deciding the best course of action, such as surgery or careful observation (78). Galectin-3 expression is used as a prognostic indication in thyroid cancer. Its overexpression is linked to an increased risk of illness recurrence and may alter postoperative care options (78). Patients with PTC who have high levels of galectin-3 may benefit from more aggressive therapy methods or diligent postoperative surveillance to detect possible recurrences early (79). Galectin-3 regulates a variety of biological functions, including cell adhesion and death. In thyroid cancer, dysregulation leads to the disruption of normal cellular activities, which promotes tumor growth (79). The specific molecular processes by which galectin-3 promotes thyroid cancer formation and progression are now being investigated, offering insights into prospective treatment targets (80). Researchers expect to uncover targets for precision medicine techniques by unraveling the complicated molecular connections involving galectin-3 (80). Recent research is helping to improve our grasp of galectin-3’s function in thyroid cancer biology and its potential implications for individualized treatment.

### 3.8 Cytokeratin 19

CK19 is an important biomarker for thyroid cancer detection, risk stratification, and prognosis (81). CK19 belongs to the cytokeratin family of intermediate filament proteins and is found in a variety of epithelial tissues, including the thyroid gland (81). In thyroid cancer, CK19 has received attention for its ability to discriminate between benign and malignant thyroid tumors (81). The thyroid gland’s epithelial cells typically express CK-19. However, its upregulation is observed in thyroid cancer, particularly PTC (81). Immunohistochemical staining for CK19 has become an important technique in pathology, assisting in the distinguishing of thyroid nodules (82). The identification of CK19 expression is especially beneficial in separating benign thyroid lesions from malignancies, allowing for more accurate preoperative diagnosis and treatment decisions (82). Elevated CK19 expression is related with more aggressive characteristics in thyroid carcinoma (83). In PTC, CK19 positive has been associated with increased tumor size, lymph node metastasis, and extrathyroidal extension (83). The presence of CK19 is used as a prognostic indication to assist identify patients who are at a higher risk of illness recurrence (84). This information assists doctors in developing postoperative treatment regimens and identifying the need for further medications or increased surveillance (84). CK19 expression aids in risk classification in thyroid cancer. Its evaluation, frequently in combination with other indicators, assists in classifying thyroid nodules into risk categories, allowing for a more tailored approach to patient care (85). High CK19 expression may impact surgical extent and the necessity for postoperative radioactive iodine therapy, giving useful information for optimizing treatment regimens (85). The upregulation of CK19 in thyroid cancer reflects molecular changes that occur in malignant thyroid cells. Understanding the molecular pathways involving CK19 sheds light on the underlying processes of tumor formation and progression (86). The importance of CK19 in maintaining cellular shape and integrity implies that it may be involved in thyroid cancer cells’ invasive characteristics (86). While CK19 is not a direct therapeutic target, its significance as a diagnostic and prognostic marker aids in the management of thyroid cancer (87). Identifying patients with high CK19 expression enables a more personalized and focused approach to therapy, stressing precision medicine tactics (87). CK19 appears as an important biomarker in the context of thyroid carcinoma (87). Its expression patterns give crucial diagnostic information, help in risk stratification, and shed light on the prognosis of people with thyroid cancer (87). The continuous investigation of CK19’s molecular contributions advances our understanding of thyroid cancer biology and may have future implications for treatment strategy refinement.

### 3.9 Circulating tumor DNA

ctDNA has emerged as a potential molecular biomarker in thyroid cancer, providing non-invasive insights into the disease’s progression (88). The term ctDNA refers to fragmented DNA shed into the circulation by tumor cells, allowing for a liquid biopsy method that has the potential to transform thyroid cancer detection, monitoring, and therapy. ctDNA is a non-invasive way to identify and monitor thyroid cancer (88). CtDNA analysis includes extracting cell-free DNA from a blood sample, which eliminates the need for invasive procedures such as conventional biopsies (88). This is particularly useful for tracking disease development and therapy response over time (88). ctDNA enables the diagnosis of little residual illness or early-stage thyroid cancer. ctDNA enables the diagnosis of little residual illness or early-stage thyroid cancer (89). Its sensitivity allows doctors to identify genetic changes linked with thyroid cancer, giving them a tool for early detection and intervention (89). ctDNA analysis involves the identification of particular genetic abnormalities, such as mutations or rearrangements. ctDNA analysis can detect prevalent genetic abnormalities in thyroid cancer, such as BRAF and RAS mutations and RET/PTC rearrangements (89). These molecular fingerprints help in tumor profiling and influence therapy recommendations. ctDNA analysis is useful for assessing therapy response and illness recurrence (90). Variations in ctDNA levels or the appearance of particular mutations during or after therapy might indicate treatment success or the need to modify therapeutic techniques (90). The dynamic nature of ctDNA enables real-time evaluation of the tumor landscape. This is especially important in thyroid cancer, where tumors can be heterogeneous, and identifying emerging genetic changes allows for more precise and focused therapy (90). ctDNA analysis gives prognostic information, which can assist predict illness recurrence or progression. Specific ctDNA patterns may suggest a greater probability of aggressive tumor activity, which might influence postoperative therapy and monitoring tactics (90). The molecular information gained from ctDNA allows for the formulation of individualized treatment methods. Targeted medicines can be tailored to particular genetic abnormalities found in ctDNA, improving the accuracy of thyroid cancer treatment (91). ctDNA is increasingly being used in clinical trials and research projects to investigate new treatments for thyroid cancer. Its involvement in finding actionable genomic targets helps to create novel treatment options and advances precision medicine (91). ctDNA is a revolutionary tool in the molecular landscape of thyroid cancer. Its non-invasive nature, ability to detect genetic alterations, and dynamic monitoring capabilities all help to improve thyroid cancer diagnosis, treatment decisions, and overall patient management (91). As technology and research advance, ctDNA is anticipated to play an increasingly important role in developing individualized and targeted therapy for thyroid cancer.

### 3.10 Programmed death-ligand 1

PD-L1 has received a lot of interest as a molecular biomarker for thyroid cancer, especially in the setting of immunotherapy (92). PD-L1 is a cell surface protein that regulates the immune response by interacting with the PD-1 receptor on immune cells (92). In thyroid cancer, PD-L1 expression influences prognosis and treatment options, particularly in the age of immune checkpoint inhibitors (92). PD-L1 is an important target in immunotherapy, especially immune checkpoint blocking. Tumors that express PD-L1 can use this route to avoid the immune system, resulting in immunological tolerance and allowing cancer cells to spread unabated (93). PD-L1 expression in thyroid carcinoma is related with a more aggressive disease progression and a worse prognosis (93). High levels of PD-L1 are frequently associated with increased tumor invasiveness, metastasis, and resistance to conventional therapies (93). The presence of PD-L1 in thyroid carcinoma is a critical factor influencing the responsiveness to immune checkpoint inhibitors (93). Tumors with high PD-L1 expression are more likely to react successfully to immunotherapy, emphasizing the value of PD-L1 testing in guiding treatment decisions (94). PD-L1 is a companion diagnostic marker for immune checkpoint inhibitor treatments. Determining PD-L1 expression levels in tumor tissues aids doctors in determining the most effective treatment approaches (94). PD-L1 expression can be dynamic, driven by a variety of variables such as the tumor microenvironment and therapeutic treatments (94). Monitoring PD-L1 levels over time provides a more nuanced knowledge of the tumor’s response to therapy and the possible formation of resistance mechanisms (95). PD-L1 status influences the development of combination therapy in thyroid cancer. Understanding the interactions between PD-L1 expression and other molecular factors can help drive the development of personalized treatment methods that combine immunotherapy with other targeted medicines (95). Present study investigates the function of PD-L1 in thyroid cancer biology. Investigating the variables controlling PD-L1 expression and its interactions with the immune system sheds light on the intricate processes that drive thyroid cancer growth and immune evasion (95). PD-L1 is a critical molecular biomarker in thyroid cancer, impacting treatment decisions and prognosis, notably in the field of immunotherapy (95). The evaluation of PD-L1 expression is critical to the developing landscape of precision medicine, assisting doctors in personalizing therapy regimens for patients with thyroid cancer (96). Future research aims to improve our understanding of PD-L1’s significance and broaden the scope of targeted and immunotherapeutic therapies in thyroid cancer.

## 4 Advances in targeted therapy of thyroid cancer

The current emphasis of thyroid cancer targeted therapy research is to find and exploit specific biochemical pathways in order to improve treatment results (97). Tyrosine kinase inhibitors (TKIs) are a popular research topic because they target critical signaling pathways involved in thyroid cancer development and progression (97). TKIs, such as lenvatinib and sorafenib, have demonstrated success in advanced thyroid malignancies, particularly those that are resistant to traditional therapies (97). Additionally, efforts are being made to study and target genetic abnormalities such as BRAF and RET changes, which are frequent in thyroid cancer (98). BRAF inhibitors, such as vemurafenib and dabrafenib, have shown potential in treating BRAF-mutant thyroid tumors (98). Immunotherapy, particularly immune checkpoint inhibitors such as pembrolizumab, is being investigated, with a focus on improving the immune response against thyroid cancer cells (98). Combining immunotherapy with additional targeted medicines is a promising method for increasing therapeutic efficacy. Precision medicine techniques based on molecular profiling are gaining traction (98). Comprehensive genetic analysis aids in the identification of particular mutations in individual tumors, allowing for the development of personalized therapy based on the unique molecular landscape of each patient’s thyroid cancer (99). Despite these advances, there are still obstacles, including as dealing with treatment resistance and unwanted effects (99). Ongoing research seeks to identify new therapeutic targets, increase therapy tolerance, and develop combination methods. The changing scientific environment offers out hope for more effective and tailored targeted medicines in the treatment of thyroid cancer (**Table 2**).

**Table 2:** Targeted therapy of thyroid cancer.

Targeted therapy	Notes	Diseases	Drugs	References
Tyrosine kinase inhibitors	Inhibiting VEGFR, FGFR, PDGFR, and RET	PTC, FTC	Lenvatinib, Sorafenib	(100–103)
BRAF inhibitors	Inhibiting BRAF	PTC	Vemurafenib, Dabrafenib	(46, 104–106)
Immunotherapy	Inhibiting PD-L1	PTC, FTC	Atezolizumab, Durvalumab	(17, 107–109)
RET inhibitors	Inhibiting RET	PTC	Selpercatinib, Pralsetinib	(110–114)
MEK inhibitors	Inhibiting MEK	PTC, FTC	Trametinib, Cobimetinib	(115–117)

### 4.1 Tyrosine kinase inhibitors

TKIs are a kind of cancer therapy that inhibits the activity of tyrosine kinases, enzymes involved in a variety of biological functions, including cell growth and division (100). In the setting of thyroid cancer, TKIs have demonstrated effectiveness in blocking particular pathways implicated in tumor development. TKIs function by suppressing the activity of tyrosine kinases, enzymes that phosphorylate tyrosine residues in proteins, a step necessary for intracellular signal transmission (100). TKIs in thyroid cancer typically target receptors such vascular endothelial growth factor receptors (VEGFR), epidermal growth factor receptors (EGFR), and other kinases involved in angiogenesis and tumor development (100). Lenvatinib targets the VEGFR, FGFR, PDGFR, and RET. It is authorized for the treatment of advanced differentiated thyroid carcinoma. Another TKI for advanced thyroid cancer patients is sorafenib, which inhibits PDGFR, RAF, and VEGFR (101). TKIs are frequently used when traditional therapies such as surgery or radioactive iodine are inadequate, or when advanced thyroid cancer cannot be physically removed (101). They are especially beneficial for aggressive thyroid tumors that have progressed outside the thyroid gland. One important feature of TKI activity is the prevention of angiogenesis, the creation of new blood vessels on which tumors rely for nutrition and oxygen (101). TKIs that target VEGFR impede the development of blood vessels, depriving the tumor of vital supplies and slowing its growth (102). TKIs may cause tiredness, hypertension, diarrhea, and skin problems. Normal monitoring is important to treat those adverse symptoms adequately (102). Eventually, certain cancers may acquire resistance to TKIs. Ongoing study seeks to identify and overcome resistance mechanisms. Combination treatments, such as TKIs combined with other targeted drugs or immunotherapy, are being investigated in order to improve therapeutic effectiveness and overcome resistance (103). The molecular features of the tumor are frequently used to choose a certain TKI. Tumor molecular profiling identifies particular genetic changes that might assist guide therapy decisions (103). Continuous research is being conducted to find novel targets and increase the efficacy of TKIs. Clinical trials are investigating novel combinations and determining their efficiency in various thyroid cancer subtypes (103). In conclusion, TKIs are an important class of targeted medicines in thyroid cancer therapy. TKIs have been shown to be beneficial in limiting tumor development and improving outcomes by specifically interfering with critical signaling pathways, especially in circumstances when standard therapies may be ineffective. Current research and clinical studies attempt to improve their efficacy, resolve resistance concerns, and expand their therapeutic potential.

### 4.2 BRAF inhibitors

BRAF inhibitors are a type of targeted therapy that inhibits the action of a mutant BRAF gene, which regulates cell growth and division (104). These inhibitors are especially important for malignancies with specific BRAF mutations, such as thyroid carcinoma (104). BRAF inhibitors work by targeting the mutant BRAF gene, most often the V600E mutation (104). This mutation results in a constitutively active BRAF protein, which contributes to uncontrolled cell proliferation (104). By blocking the action of mutant BRAF, these inhibitors hope to disrupt the signaling cascade known as the MAPK/ERK pathway, which is abnormally active in cells with the V600E mutation (105). Vemurafenib and Dabrafenib are both well-known BRAF inhibitors (105). Vemurafenib was first designed for melanoma, and it has also been used to treat thyroid carcinoma with BRAF mutations (105). Dabrafenib is another BRAF inhibitor used to treat BRAF-mutant thyroid carcinoma (105). BRAF inhibitors are mostly utilized in thyroid cancer patients with the BRAF V600E mutation (46). They are commonly used when standard therapies, such as surgery and radioactive iodine, are ineffective or in situations of advanced thyroid cancer (46). BRAF inhibitors have demonstrated considerable effectiveness in lowering tumor size and slowing disease progression in BRAF-mutant thyroid malignancies (46). Response rates differ amongst individuals, and the length of response may be impacted by factors such as the existence of other genetic abnormalities (106). Combining BRAF inhibitors with other targeted medicines or immunotherapies is an ongoing research topic. Combinations are intended to improve treatment results, overcome resistance, and address the heterogeneity of thyroid cancer (106). Common side effects of BRAF inhibitors include skin problems, fatigue, fever, and joint pain. Monitoring for side effects and adjusting treatment as necessary are critical components of patient care. Some cancers may become resistant to BRAF inhibitors over time (106). Ongoing research aims to better understand the processes of resistance and find solutions to overcome this issue. Clinical studies for new BRAF inhibitors and combination therapy are now underway, adding to the developing landscape of precision medicine in thyroid cancer (106). Molecular profiling of tumors is critical for identifying individuals with BRAF mutations who might benefit from BRAF inhibitors. Patient selection based on genetic features improves therapy outcomes (106). To summarize, BRAF inhibitors are an effective targeted treatment for particular subtypes of thyroid carcinoma with BRAF mutations. These inhibitors, which directly block the aberrantly active BRAF protein, have showed promise in limiting tumor development. Ongoing research seeks to improve their usage, address resistance mechanisms, and investigate combination tactics to increase their efficacy in treating thyroid cancer.

### 4.3 Immunotherapy

Immunotherapy is a novel method to cancer treatment that uses the immune system of the patient to identify and remove cancer cells (17). Immunotherapy has showed promise in the treatment of thyroid cancer, especially in advanced instances where standard therapies may be ineffective (17). Immune checkpoint inhibitors are important components of immunotherapy for thyroid cancer. These medications target particular proteins on immunological or cancer cells, altering inhibitory signals that keep the immune system from successfully targeting cancer cells (17). Pembrolizumab and nivolumab are PD-1 inhibitors, whereas atezolizumab and durvalumab are PD-L1 inhibitors. They have been examined in several malignancies, including thyroid carcinoma (107). Tumors can use immunological checkpoints, such as PD-1/PD-L1, to avoid immune detection. Immunotherapy suppresses these checkpoints, allowing T cells to better detect and destroy cancer cells (107). Immunotherapy is often used in situations of advanced thyroid cancer that has not responded well to conventional treatments such as surgery, radioactive iodine, or targeted therapies (107). PD-1/PD-L1 inhibitors have shown benefit in certain types of thyroid cancer patients, notably those with poorly differentiated or anaplastic thyroid carcinoma (107). Immunotherapy has shown long-term responses in certain patients, resulting in tumor reduction and increased overall survival (108). Immunotherapy responses might vary, and ongoing research is aimed at identifying predictive indicators to help select individuals who would benefit (108). Combinations of immunotherapy with other targeted treatments, chemotherapy, or radiation are being investigated in order to improve treatment results and overcome resistance mechanisms. While immunotherapy is typically well tolerated, it can cause immune-related side effects such as tiredness, skin rash, and organ inflammation (108). Prompt detection and control of these adverse effects is critical to patient safety. Biomarker testing, such as PD-L1 expression, is frequently used to identify individuals with a higher likelihood of responding to immunotherapy (109). However, reactions can still occur in people who have minimal or no PD-L1 expression (109). To summarize, immunotherapy represents a game-changing technique in the treatment of thyroid cancer. It provides new hope to patients with advanced or resistant illness by releasing the immune system’s strength. Ongoing research aims to improve its usage, broaden its application to diverse subtypes, and increase immunotherapy’s overall success in the treatment of thyroid cancer.

### 4.4 RET inhibitors

RET inhibitors are a type of targeted therapy that interferes with the action of the RET protein, notably in malignancies with RET mutations or fusions (110). These inhibitors have demonstrated effectiveness in inhibiting the aberrant signaling associated with RET-driven malignancies (110). RET is a receptor tyrosine kinase that regulates cell development and differentiation (110). In tumors with RET changes, such as rearrangements or mutations, the RET signaling system is abnormally active (110). RET inhibitors work by targeting the RET protein or disrupting downstream signaling pathways to prevent cancer cells from growing and dividing uncontrollably (111). Selpercatinib is authorized for a variety of malignancies with RET mutations, including several kinds of thyroid carcinoma (111). Pralsetinib is another RET inhibitor utilized to treat tumors with RET mutations (111). RET inhibitors are generally utilized in malignancies where RET mutations are detected by molecular testing (111). These changes can occur in both papillary and medullary thyroid tumors (112). RET inhibitors have shown effectiveness in reducing tumor development and inducing significant responses in individuals with RET-altered malignancies (112). Patients’ responses may vary, and continuing study strives to better understand the elements that influence therapy results (112). Clinical trials are looking into the potential benefits of combining RET inhibitors with other targeted medicines or immunotherapy to improve treatment efficacy and address resistance mechanisms (112). Fatigue, hypertension, gastrointestinal difficulties, and changes in liver enzyme levels are some of the most common RET inhibitor adverse effects. Regular monitoring and control of side effects are critical components of patient care (113). Resistance to RET inhibitors can develop over time, and ongoing research seeks to uncover and overcome these pathways (113). Further research is being conducted to determine the appropriate therapy sequence and the feasibility of combining RET inhibitors with other treatment methods (113). Molecular profiling of cancers is critical for identifying individuals with RET mutations who might benefit from RET inhibitors (113). Targeted medicines are most successful when they are customized to the unique genetic features of each tumor (114). In conclusion, RET inhibitors offer a viable treatment alternative for malignancies caused by RET mutations, including some kinds of thyroid cancer. These inhibitors have been shown to be effective in reducing tumor development and improving outcomes by addressing the underlying molecular abnormalities. Ongoing research is required to improve their usage, address resistance concerns, and investigate combination tactics to increase their efficacy in treating malignancies with RET mutations.

### 4.5 MEK inhibitors

The MAPK pathway is a signaling route that regulates cell growth, differentiation, and survival (115). MEK inhibitors are a kind of targeted therapy that interferes with the action of MEK (MAPK/ERK kinase), a critical enzyme in this pathway (115). These inhibitors are especially useful in malignancies when the MAPK pathway is dysregulated (115). MEK inhibitors work by selectively targeting MEK, which phosphorylates and activates ERK in the MAPK pathway (116). By inhibiting MEK, these inhibitors impair the downstream signaling cascade, affecting cell growth and proliferation (116). Trametinib is licensed for the treatment of certain malignancies with BRAF mutations and is frequently used in conjunction with BRAF inhibitors (116). Cobimetinib is another MEK inhibitor that is utilized in conjunction with BRAF inhibitors in some malignancies, including melanoma (116). MEK inhibitors are typically utilized in malignancies characterized by MAPK pathway dysregulation, which is frequently caused by mutations in BRAF or other pathway components (117). They are frequently used in conjunction with BRAF inhibitors in malignancies with BRAF mutations, such as melanoma and some kinds of thyroid cancer (117). MEK inhibitors, particularly when combined with BRAF inhibitors, have been found to effectively suppress tumor development and improve outcomes in some malignancies (117). Responses can differ between individuals, and ongoing research tries to uncover characteristics that influence therapy results (117). Combining MEK inhibitors with other targeted treatments, immunotherapy, or chemotherapy is an important research topic to improve therapeutic efficacy and overcome possible resistance mechanisms. MEK inhibitors may cause skin problems, gastrointestinal discomfort, exhaustion, and changes in blood cell counts. Regular monitoring and control of adverse effects is required (117). Resistance to MEK inhibitors can develop, necessitating continued research to identify and overcome these resistance pathways (117). Investigational studies investigate the use of MEK inhibitors in various combinations and sequencing to maximize treatment options (118). Molecular screening of malignancies is critical for identifying individuals with particular mutations or dysregulation in the MAPK pathway who may benefit from MEK inhibitors (118). Personalized therapy options are critical for maximizing therapeutic outcomes (118). In summary, MEK inhibitors are an important class of targeted treatments, especially in malignancies with dysregulated MAPK pathway signaling. These inhibitors have shown effective in suppressing tumor development by interfering with this essential mechanism. Future research aims to improve their usage, address resistance mechanisms, and investigate combination tactics to boost their efficacy in a variety of malignancies.

## 5 Conclusion and perspectives

The progress of targeted therapy and biomarker research in thyroid cancer represents a possible paradigm change in therapeutic methods, aimed at precision and individualization. Here are some recommendations for investigating targeted therapy and biomarker studies in thyroid cancer: (1) Improve molecular diagnosis and tailored therapy. Advocating for the wider use of molecular diagnostic tools in thyroid cancer patients, such as genetic mutation analysis, protein expression profiling, and biomarker identification. Promoting the use of thorough molecular profiling, which includes identification of common mutations such as BRAF, RET, and RAS, to accurately guide the selection of appropriate targeted therapy. Teaching healthcare personnel how to create individualized treatment regimens based on a patient’s molecular traits and biomarker data. (2) Promote multi-center collaborative research projects. Supporting the formation of multi-center collaborative research initiatives that allow for the sharing of patient samples and clinical data, therefore speeding our understanding of biomarkers and targeted therapeutics in thyroid cancer. Encourage cross-institutional and worldwide collaboration to expand study sample sizes, resulting in more compelling and therapeutically relevant research outputs. (3) In-depth research on critical targets such as BRAF and RET. Intensifying research into important molecular targets such as BRAF and RET to better understand their involvement in thyroid cancer development. Driving research into new and more effective BRAF and RET inhibitors, as well as developing novel techniques to potentially reduce therapy resistance. (4) Use of bioinformatics and artificial intelligence in biomarker analysis. Increasing the use of bioinformatics and artificial intelligence technology in biomarker research to decipher complicated biological interaction networks and predict treatment outcomes. Using big data analytics to identify possible new biomarkers, resulting in more complete information for thyroid cancer therapy. (5) Investigate the possibilities of immunotherapy. Increasing research examines the possibilities of immunotherapy in thyroid cancer, includes the study of immune-related indicators including PD-L1 expression and tumor-infiltrating lymphocytes (TILs). Driving clinical studies to study the combination of immunotherapy and targeted treatments in order to improve therapeutic effectiveness. (6) Concentrate on the issue of treatment resistance and recurrence. To develop more successful treatment techniques, researchers are looking at treatment resistance mechanisms, including as recurrence patterns following targeted therapy and immunotherapy. Promoting the start of long-term follow-up studies to better understand post-treatment survival outcomes and the quality of life of thyroid cancer patients. (7) Patient education and engagement. Emphasizing the necessity of patient education about the advantages and potential negative effects of targeted therapy, as well as the value of biomarker testing. Encouraging patient participation in clinical trials, creating a collaborative approach to research, and enhancing treatment alternatives. In conclusion, these guidelines highlight the need of a holistic and collaborative approach to thyroid cancer research, utilizing advances in molecular diagnostics, targeted therapeutics, and biomarker analysis. Such activities are crucial for increasing precision medicine in thyroid cancer treatment and improving patient outcomes.

## Author contributions

MG: Conceptualization, Funding acquisition, Software, Writing – original draft, Writing – review & editing. YS: Conceptualization, Writing – original draft. YW: Conceptualization, Writing – original draft. JX: Conceptualization, Writing – original draft. CZ: Funding acquisition, Software, Validation, Writing – review & editing.
